# CRISPR–Cas9 a boon or bane: the bumpy road ahead to cancer therapeutics

**DOI:** 10.1186/s12935-019-0726-0

**Published:** 2019-01-08

**Authors:** Debarati Ghosh, Prabhadevi Venkataramani, Saikat Nandi, Sonali Bhattacharjee

**Affiliations:** Cold Spring Harbor Laboratory, Cold Spring Harbor, NY USA

**Keywords:** Gene editing, CRISPR–Cas9, Targeted cancer therapy, Precision medicine, CAR-T therapy, Tumor heterogeneity, Drug resistance, Immunotherapy, Clinical trials

## Abstract

Genome editing allows for the precise manipulation of DNA sequences in a cell making this technology essential for understanding gene function. CRISPR/Cas9 is a targeted genome-editing platform derived from bacterial adaptive immune system and has been repurposed into a genome-editing tool. The RNA-guided DNA endonuclease, Cas9 can be easily programmed to target new sites by altering its guide RNA sequence, making this technology easier, more efficient, scalable and an indispensable tool in biological research. This technology has helped genetically engineer animal models to understand disease mechanisms and elucidate molecular details that can be exploited for improved therapeutic outcomes. In this review, we describe the CRISPR–Cas9 gene-editing mechanism, CRISPR-screening methods, therapeutic targeting of CRISPR in animal models and in cancer immunotherapy. We also discuss the ongoing clinical trials using this tool, limitations of this tool that might impede the clinical applicability of CRISPR–Cas9 and future directions for developing effective CRISPR–Cas9 delivery systems that may improve cancer therapeutics.

## Introduction

Cancer is the second leading cause of mortality worldwide accounting for 8.8 million deaths in 2015 (WHO). Globally, 1 out of 6 death is caused by cancer (WHO). The cost associated with cancer is significant and increasing with an estimated annual cost of approximately USD 1.16 trillion in 2010 [1]. The socio-economic burden and mortality associated with cancer is largely due the gaps in our understanding of the molecular details of the disease and lack of cost effective treatment regimens. Adding to this complexity are the issues of differential mutational load, tumor heterogeneity and therapeutic resistance that determine clinical outcomes for targeted cancer therapies. The gene editing technology, CRISPR/Cas9 has allowed us to better understand how a gene product contributes to development and disease in an organism. In this review, we discuss developing effective CRISPR–Cas9 delivery systems for improved cancer treatment.

## CRISPR/Cas system: a gene-editing tool

Understanding a gene function relies on controlled alteration of its DNA sequence in a cell. Although, several editing enzymes like zinc finger nucleases (ZFNs), transcription activator-like effector nucleases (TALENs) and homing meganucleases are effective they require reengineering the enzyme for each target sequence [2–4]. On the other hand, homologous recombination (HR) based genetic engineering methods have lower editing efficiency and thereby warrant screening a larger sample size [5]. The clustered regularly interspaced short palindromic repeats (CRISPR)/CRISPR-associated nuclease 9 (Cas9) is a technology which is scalable, affordable and easy to engineer [6, 7]. The CRISPR/Cas9 system was first observed as microbial defense immunity against invading viruses or other genetic elements [8, 9]. This natural system was further adapted for genome editing by programming site-specific DNA double strand breaks (DSBs) and editing the mammalian genome with high precision [10].

**Fig. 1:**
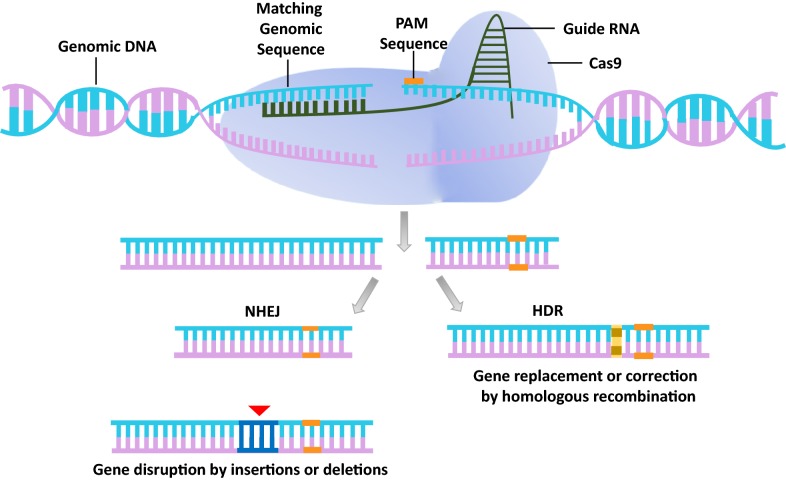
Schematic of CRISPR–Cas9-mediated genome editing. The *Streptococcus pyogenes* derived CRISPR–Cas9 RNA-programmable DNA endonuclease is targeted to a DNA sequence via a single guide RNA (sgRNA) sequence, which base-pairs with a 20-nt DNA sequence upstream of the protospacer-associated motif (PAM), resulting in a 3-bp double-strand break (DSB) upstream of the NGG. The resulting DSBs are subsequently repaired either by non-homologous end joining (NHEJ) or by homology-directed repair (HDR). Repair via the error-prone NHEJ pathway, frequently leads to insertion or deletion mutations (Indels) that can lead to genome instability. Alternatively, in the presence of an exogenous donor DNA template, the DSB can be repaired via the error-free HDR pathway, which can engineer precise DNA modifications

Cas9 generates DSBs at a target genetic locus like ZFNs [11–14] and TALENs [15–21]. The advantage of Cas9 editing is that its nuclease function is guided either by a natural dual-RNA complex or a chimeric single-guide RNA (sgRNA) that recognizes target sequences via Watson–Crick base pairing [22, 23]. The DSB generated by the nuclease action of Cas9 activates the cellular DNA damage response that is critical for the maintenance of genome stability [24]. Organisms have evolved with two major pathways for the repair of DSBs in mammalian cells: the non-homologous end joining (NHEJ) pathway, which is a template-independent error prone pathway of DNA double strand break repair (DSBR) and the homology directed repair (HDR) pathway which relies on extensive homology for DSBR and is considered a error free pathway [25–29]. Repair by NHEJ introduces insertion/deletion (Indel) mutations and larger deletions in the genome [30–32]. In contrast HDR, introduces a site-specific mutations at a target locus [33] (Fig. 1).

There is an increasing effort in re-engineering the CRISPR/Cas system in order to meet three major aims: (i) decreasing size of the Cas9 nuclease (ii) increasing its fidelity and (iii) improving Cas targeting efficiency [34].

Three types (I–III) of CRISPR systems have been identified thus far, each system contains (i) cluster of CRISPR-associated (Cas9) genes, (ii) non-coding RNAs and (iii) an array of repetitive elements (direct repeats) [35]. The direct repeats interspersed by protospacers (short variable sequences derived from the exogenous DNA targets) forms the CRISPR RNA (crRNA) array [35]. Each CRISPR system contains a protospacer adjacent motif (PAM) within the DNA target [8, 36, 37]. The CRISPR–Cas system is divided into two classes: class 1 contains multiple Cas proteins, and class 2 contains a single Cas protein [38–40] (Table 1). Class 1 system is composed of types I, III and IV and the class 2 CRISPR/Cas system is further divided into types II, V, VI [41]. The type I systems use Cas3 enzyme, type II systems use Cas9, type III systems use Cas10 enzyme, type IV systems use Csf1 protein, type V systems use Cpf1, C2c1 or C2c3, and type VI use protein Cas13a [42]. The best-characterized CRISPR system is the type II CRISPR system; it consists of the Cas9 nuclease, crRNA array and an additional trans-activating crRNA (tracrRNA) [22, 23, 43]. The crRNA and tracrRNA are fused to form a chimeric sgRNA. By altering this 20nt guide sequence within the sgRNA, Cas9 can be redirected to any target sequence in the vicinity of the PAM sequence, typically NGG [43]. The type-II Cas9 effector nucleases are best characterized and are used for genome engineering technologies [44, 45].

**Table 1:** Cas9 proteins and their function in CRISPR editing

Cas protein	CRISPR system	Guide RNA	Nuclease activity	Recognition sequence on target	Reference
Cas9	Type II	crRNA + transcrRNA	Yes	G-rich PAM	[49, 50]
Cpf1	Type V	crRNA	Yes	G-rich PAM	[46]
Cas13a	Type VI-A	crRNA + transcrRNA	Yes	Except G-rich	[54]
dCas9	Type II	crRNA + transcrRNA	No	G-rich PAM	[140]
Cas3	Type I	crRNA	Yes	T-rich PAM	[141, 142]
Cas10/Csm1	Type III	crRNA	Yes	AT-rich PAM	[143, 144]
dCas13a	Type VI-A	crRNA + transcrRNA	Yes	Except G-rich	[145]

A new Cas effector nuclease Cpf1 (also known as Cas12a) has been recently identified [46]. It is a type V effector and is guided by crRNA rather than crRNA and trancrRNA dual guide system used by Cas9 [46, 47]. Also, Cpf1 recognizes T-rich PAM sequences adjacent to the target DNA unlike Cas9, which recognizes G-rich PAM [43, 48–50]. Furthermore, Cpf1 introduces a staggered DSB with a 4 or 5-nt overhang at the PAM-distal region unlike Cas9, which generates blunt ends at its PAM-proximal region [23]. Cpf1 shows better target specificity than Cas9 [51, 52] and can also process its own crRNA array to generate mature crRNAs [53].

One screening study using 15 orthologous has lead to the identification of another nuclease, Cas13a from *Leptotrichia wadei* (LwaCas13a) [54]. LwaCas13a has shown to be effective in targeted knockdown of reporter or endogenous targets to similar levels as RNA interference but with greater specificity [54].

Biochemical and genetic studies demonstrate effector nuclease Cas13b, which can process its own CRISPR array with short and long repeats, has RNase activity and can cleave target RNA [55]. It also has a protospacer-flanking sequence required for RNA targeting [55]. The associated proteins Csx27 represses, whereas Csx28 enhances the Cas513b mediated RNA interference [55].

Mutating both nuclease domains of Cas9 renders the protein catalytically dead (dCas9). dCas9 can bind the target DNA without cleaving it [56, 57]. The dCas9 fusion protein [Cas9-KRAB] with Kruppel associated box (KRAB) domain of Kox1 protein recruits chromatin modifying factors and silences gene expression [57]. dCas9 can also be used for gene activation, this approach is known as CRISPR activator (CRISPRa); dCas9 can also mediate reversible CRISPR interference (CRISPRi) [57–60]. Gene editing can be modulated by combining CRISPRi and CRISPRa [61].

## CRISPR-screening

High throughput gain-of-function (GOF) or loss-of-function (LOF) screening is important for identifying new factors in a pathway or interactions between components of a pathway [62]. Limitations of the conventional short hairpin RNA (shRNA) mediated gene silencing include off target effects, high false positive rates, high false negative rates and lack of high throughput data due to high costs and large library sizes [63–66]. On other hand, cDNA and ORF mediated GOF screens are high throughput, but time consuming and cannot be used for genome-wide studies due to technical limitations [67]. sgRNA mediated screens are most useful for high throughput screening because the short and uniform sgRNA size allows for targeting the whole genome [68, 69]. In cancer, CRISPR screens have helped identify genes involved in drug resistance [60, 70–72], synergistic and synthetic lethal interactions [73, 74], regulators of PD-L1 expression [75] and others [76, 77].

### Loss-of-function CRISPR library screen

CRISPR knock out and CRISPRi has been successfully used to screen large sgRNA libraries. This approach has identified essential cancer genes implicated in cell signaling, differentiation, survival and regulatory processes [76–78]. The best examples of the success of this screening method include the identification of the role of BCR and ABL as lethal hits in chronic myelogenous leukemia (CML) cell line KBM7, KRAS and PIK3CA and as lethal hits in colorectal cancer cell lines DLD-1 and HCT116 [68, 76]. KRAS-mutated DLD-1 cell line showed high dependency on mitochondrial function [76]. Burkitt’s lymphoma cell line Raji showed dependency on the DEAD-box helicase DDX3Y located on the Y-chromosome [77].

On the other hand, positive selection screening with LOF CRISPR libraries have provide crucial information regarding drug resistance [68, 69]. A drug resistant screen performed on KBM7 cells against antimetabolite 6-thiogranine identified genes involved in DNA mismatch repair [79]. Another screen used DNA topoisomerase II (TOP2A) poison etoposide to identify TOP2A gene and cyclin-dependent kinase 6 (CDK6) in promyelocytic leukemia (PML) cell line HL60 [68]. A drug resistance screen with therapeutic RAF inhibitor vemurafenib in melanoma cell line A375 showed involvement of well characterized genes like NF1 and MED12 as well as novel hits like NF2, CUL3, TADA2B and TADA1 [69].

### Gain-of-function CRISPR library screen

The GOF screens aim at identifying positive and negative regulators of cancer proliferation pathways and use CRISPRa as the screening method [60]. CRISPRa mediated activation of tumor suppressors and lineage differentiation genes in K562; a CML cell line showed inhibition of cell proliferation [80]. This suggests a transient inactivation of the tumor suppressor genes during cell proliferation. CRISPRa mediated activation of drug resistance genes for the BRAF inhibitor vemurafenib in A375 cells suggests a mechanism of bypassing BRAF inhibition by either reactivating the MAPK pathway through BRAF independent mechanisms or MAPK-independent parallel pathways [60].

### In-vivo CRISPR screening

While in vitro screens answer some important questions about the intrinsic properties of cancer cells and potential therapeutic opportunities, they have failed to answer some key questions [81]. For example, how does the complex interaction between the cell and its microenvironment influence tumor behavior? In vivo CRISPR screens are performed to answer these questions. The first in vivo CRISPR screen was designed to investigate the role of annotated genes in tumor growth and metastasis when mutagenized [82].

#### Transplant based in vivo CRISPR screens

In vivo CRISPR screen is a two-step workflow: (i) introduction of sgRNA library to cells in culture systems and (ii) transplantation into mice to assess phenotypes in vivo [83]. The cells are sequenced after selection to identify reduced and/or depleted sgRNAs [84]. Several in vivo CRISPR screens have lead to the identification of tumor suppressors, oncogenes, synthetically lethal genes, metastasis and regulators of cancer immunotherapy in co-culture systems and transplant tumor models [82, 84–89]. However, transplant based in vivo CRISPR screens have certain downsides; for example: (i) large number of cancer cells in mice do not mimic in vivo condition, (ii) sub-cutaneous transplantation does not reflect the same result as orthotropic transplantation to relevant organ, and (iii) grafting in immunodeficient mice does not allow for the study of cancer-immune interactions.

#### Direct in vivo CRISPR screen

Direct in vivo CRISPR screens overcome some of limitations of transplant based in vivo CRISPR screens because in vivo mutagenesis is performed directly at the organ site [90, 91]. Multiplexed mutagenesis in hepatocytes of Cas9 mice has been reported when sgRNA containing plasmids are injected into tail vein [92]. A lentivirus mediated sgRNA delivery into lung intratrachea leads to mutagenesis in Cas9 expressing lung epithelial cells [93]. Furthermore, direct mutagenesis in mouse brain is made possible by using adenoassociated viruses (AAVs) [94]. A CRISPR/Cas9 mediated multiplexed-mutagenesis has been recently performed to induce hepatocellular carcinoma (HCC) and intrahepatic cholangiocarcinoma (ICC) showing that this approach is suitable for both recessive genetic screening and high-throughput cancer gene validation in mice [95]. An AAV sgRNA library containing 280 sgRNAs targeting 56 genes delivered into mouse brain induced glioblastoma mimicking the pathology of the human disease [90, 96]. However, direct in vivo CRISPR screening approaches also have its own limitations which include (i) unknown cell–cell interactions in the host tissue, (ii) low viral transduction efficiency, and (iii) immune rejection [90].

## Therapeutic targeting of CRISPR in cancer and animal models

**Fig. 2:**
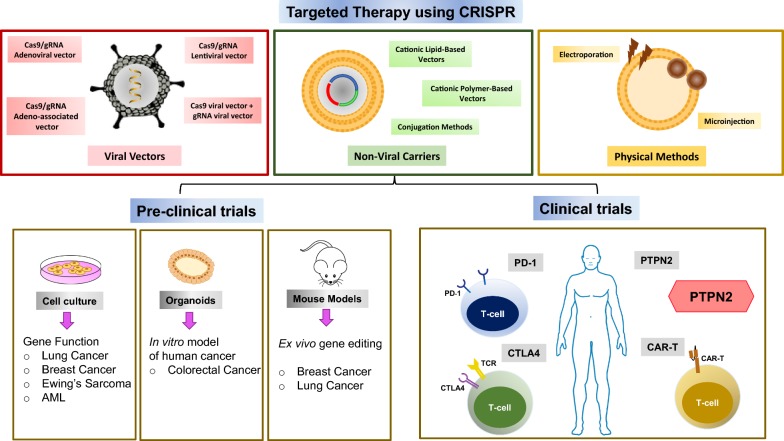
Application of CRISPR–Cas9-mediated genome engineering in studying preclinical models as well as in editing clinical targets

Along with high throughput screening, CRISPR/Cas9 mediated editing has been used to generate disease models which can be used for preclinical validation of oncogenes, drug targets and drug resistance in cancer (Fig. 2).

### Cell lines

CRISPR/Cas9 and TALEN mediated knock-in of inducible degron tags (Degron KI) allow for the specific, inducible and allele specific inhibition of protein function [97]. This approach has provided a better understanding of the pharmacological EZH2 and PI3Kα inhibitors in cancer cell lines [97]. The Degron-KI system has also helped identify the link between putative oncogene SF3B1 hotspot mutations and splicing alterations [97]. CRISPR/Cas9 has been used to study genomic rearrangements including CD74-ROS1 translocation and EML4-ALK and KIF5B-RET inversion events, which are drivers of lung cancer [98]. CRSIPR/Cas9 mediated genome engineering has also been used to generate cell lines carrying chromosomal translocations which provide information about early events in the pathology of acute myeloid leukemia (AML) and Ewing’s sarcoma [99]. CRSIPR/Cas9 mediated genome engineering has also been used to generate cell lines, followed by a second screen in vivo in order to identify novel targets for AML therapy [100]. CRISPR mediated editing of the HER2 gene in breast cancer cell lines revealed a novel mechanism of anti-cancer effects of HER2 targeting by CRISPR/Cas9, an alternative to clinical drug Herceptin [101].

### Organoid cancer models

CRISPR/Cas9 mediated editing has been used to introduce multiple mutations in human intestinal epithelium derived organoids [102]. Isogenic organoids with mutations in tumor suppressor genes APC, SMAD4, TP53 and oncogenes KRAS and/or PIK3Ca are selected and implanted in mice where they form tumors and successfully mimic colon cancer in vitro [103]. CRISPR/Cas9 mediated deletion of DNA repair genes in colon organoids has been successfully modeled mismatch repair deficient colorectal cancer [104]. CRISPR–Cas9-based genome editing of PDAC driver genes in pancreatic tumor organoids reveals Wnt niche independence during tumorigenesis [105].

### Mouse models

CRISPR/Cas9 driven mutagenesis has been used to mutate 5 genes (Tet1, 2, 3, Sry, Uty-8 alleles) in mouse ES cells in a single step, which reduces the complexity of generating multi-gene cancer models [106]. Tet1 and Tet2 gene mutations were generated with 80% efficiency by coinjecting Cas9 mRNA and sgRNAs directly into the mice zygote [106]. Cre-dependent somatic activation of oncogenic Kras (G12D) in combination of CRISPR/Cas9 mediated genome editing led to the generation of lung adenocarcinomas in mouse models which enabled the functional characterization of putative cancer genes [93]. A hydrodynamic injection of CRISPR plasmid DNA expressing Cas9 and sgRNAs against tumor suppressor genes Pten and p53 alone or in combination directly into the mouse liver induced tumor and is being used as a cancer model [92]. A point mutation in Ctnnb1 gene in the liver was introduced by hydrodynamic injection of Cas9 plasmid and Ctnnb1 sgRNA together with a mutant Ctnnb1 HDR template [92]. The intratracheal delivery of adenovirus or lentiviral vector with Cas9 and sgRNA for Em14 and Alk led to tumor formation in mouse lungs [107, 108].

Cre dependent Cas9 knock in mouse was generated and using these mice, KRAS, p53 and LKB1 gene mutations were modeled for lung adenocarcinoma [109]. An intraductal injection of lentivirus-encoding Cas9 was used to generate invasive lobular breast carcinoma (ILC) with conditional alleles of Cdh1 gene, encoding E-cadherin [110, 111].

## CRISPR in cancer immunotherapy

Immunotherapy is increasingly becoming the cornerstone of current day cancer treatment [112]. The use of immune checkpoint inhibitors in immunotherapy has been shown to reverse dysfunctional or exhausted T-cells and thereby improve efficacy in solid and hematological cancers [113, 114]. A second line of immunotherapy involves the use of genetically engineered T-cells such as the chimeric antigen receptor (CAR-T) [115] (Fig. 2).

### CRISPR/Cas-9 in CAR-T

In this approach, patient derived T-cells are genetically modified in vitro and then introduced back in patients in order to improve the efficacy of targeted T-cells killing [116–120]. CAR has an intracellular chimeric signaling domain, which helps activate T-cells, and also has a single-chain variable fragment that specifically recognizes tumor antigens [121, 122]. So far, CAR-T cell therapy has been most successful in targeting CD19 surface marker in B cell malignancies due to its expression pattern in this cellular subtype [123, 124]. Other limitation of CAR-T cell therapy includes, (i) generation of patient derived CAR-T cells (autologous CAR-T cells) is costly, (ii) time consuming and (iii) technically challenging [125]. In order to overcome these problems, allogeneic universal T-cells are derived from healthy donors, which do not contain endogenous TCR and HLA class I to eliminate graft-versus-host rejection [126]. CRISPR/Cas9 has been used to disrupt multiple loci to generate universal CAR-T donor cells [127]. The Fas receptor (CD95), upon combining with its ligand, leads to induction of T-cells apoptosis and CAR-T function. Therefore, Fas knockout CAR-T cells generated by CRISPR/Cas-9 show improved tumor cell death and prolonged survival in mice [127]. Furthermore, CRISPR/Cas9 mediated generation of CAR into the T-cell receptor alpha constant (TRAC) locus showed an uniform CAR expression in T-cell, increased T-cell efficiency and retention in mouse model of AML [128].

### CRISPR/Cas9 in PD-1 and CTLA-4

The CRISPR/Cas9 based editing has been used to disrupt T-cell surface receptors, such as programmed cell death protein 1 (PD-1) and cytotoxic T lymphocyte-associated protein 4 (CTLA-4) in order to increase efficiency of T-cell based immunotherapy [127, 129, 130]. The first clinical trial used CRISPR/Cas9 mediated PD-1 knockout T-cells in lung cancer patients [131]. Ongoing clinical trials include PD-1 knockout autologous T-cells in prostate cancer (NCT02867345), bladder cancer (NCT02863913) and renal cell carcinoma (NCT02867332). CRISPR/Cas-9 mediated simultaneous knockout of 4 loci of PD-1 and CTLA-4 have been successful in generating allogeneic universal T-donor cells [127]. CRISPR/Cas9 mediated generation of lymphocyte activating gene-3 (LAG-3) knockout CAR-T cells shows better specificity and anti-tumor potential in xenograft mouse models [132].

### CRISPR/Cas-9 and PTPN2

Immunotherapies with PD-1 checkpoint inhibitors do not work for majority of patients. This necessitates identifying alternative targets for immunotherapy. A CRISPR–Cas9 based in vivo screen identified protein tyrosine phosphatase non-receptor type 2 (PTPN2) as a novel immunotherapy target in cancer [85]. The study showed enhanced efficacy of immunotherapy by deletion of PTPN2 via interferon gamma mediated pathway [81].

## Clinical trials

CRISPR/Cas9 mediated cancer gene editing was first tested in patients with aggressive lung cancer (NCT02793856). In this trial, the immune cells from recipient blood was removed followed by ex vivo CRISPR/Cas9 editing thereby disabling PD-1 protein [131]. In vivo CRISPR/Cas9 genome editing is ongoing and shows promise but is yet to be tested in clinical trails. A new clinical trail (NCT03057912) proposes to use a combination of TALENs and CRISPR/Cas9 in the treatment of HPV-related cervical neoplasma by targeting HPV16 and HPV18 E6/E7 DNA; this approach promises to reduce off-target effects. Table 2 summarizes a comprehensive list of ongoing CRISPR/Cas9 clinical trials.

**Table 2:** Clinical trials studies on CRISPR/Cas9 in different type of cancer

Target	Cancer type	Stage of testing	Reference
CISH gene within tumor-infiltrating lymphocytes inactivated by CRISPR/Cas9	Metastatic gastrointestinal epithelial cancer	Phase II	NCT03538613
PD1 knockout engineered T- cells	Advanced esophageal cancer	Phase II	NCT03081715
PD-1 knockout EBV-CTLs	Advanced stage Epstein–Barr virus (EBV) associated malignancies	Phase II	NCT03044743
Gene-disrupted allogeneic CD19-directed BBζ CAR-T cells (UCART019)	Relapsed or refractory CD19+ leukemia and lymphoma	Phase II	NCT03166878
Dual Specificity CD19 and CD20 or CD22 CAR-T Cell Immunotherapy	Relapsed or refractory leukemia and lymphoma	Phase II	NCT03398967
PD-1 and TCR gene-knocked out mesothelin-directed CAR-T cells	Mesothelin positive multiple solid tumors	Phase I	NCT03545815
PD-1 knockout engineered T Cells	Metastatic non-small cell lung cancer	Phase I	NCT02793856
NY-ESO-1-redirected CRISPR (TCRendo and PD1) edited T-cells (NYCE T-cells)	Multiple myeloma melanoma synovial sarcoma myxoid/round cell liposarcoma	Phase I	NCT03399448

## Challenges of CRISPR/Cas9 in therapeutic targeting

CRISPR/Cas9 mediated in vitro and in vivo gene editing is an excellent tool for studying disease mutations. However, it is not error-free and the system fails about 15% of the time [133–135]. Below we summarize the major challenges of CRISPR/Cas9 mediated cancer therapy.

### Cas9-DSB complex

Recent studies have shown that the persistent binding of Cas9 to DSBs blocks the access of repair proteins to the break site, thereby reducing repair efficiency [136]. This makes Cas9-DSB complex the rate-limiting step during genome editing in vivo. Studies have also shown that template bound Cas9 can be dislodged by the translocating RNA polymerase only when the polymerase approaches the DSB from a particular direction [129].

### Immunity against Cas9 protein

A potential explanation for the failure of CRISPR/Cas9 mediated editing in vivo arises from the humoral and cell-mediated adaptive immune response to bacterial Cas9 [137]. A recent study aimed at finding the presence of pre-existing adaptive immune response to the Cas9 homologs: SaCas9 (*S. aureus* homolog of Cas9) and SpCas9 (*S. pyogenes* homolog of Cas9). This study reported the presence of 79% of donors stained positive for SaCas9 and 65% of donors stained positive for SpCas9. Also, in peripheral human blood, 46% donor stained for SaCas9 T-cells [137].

### On-target effects of CRISPR/Cas9 editing

Using long-read sequencing and long-range PCR genotyping, a recent study revealed that repair of DNA breaks introduced by Cas9 result in large deletions and genetic alterations [138]. The authors also observed that both the lesions and the resulting crossover event occur far from the cut site [138]. This study brings forth the issue of CRISPR/Cas9 mediated on-target damage, which may lead to activation of dormant oncogenes, inactivation of tumor-suppressors genes and other disease causing genes [138]. The on target effects of CRISPR/Cas9 can be avoided by sequencing a few clones and looking for unwanted genomic alteration before introducing the clones back for expansion [138].

### Off-target effects of CRISPR/Cas9 editing

Although, the targeting efficiency of Cas9 is controlled by the first 20-nucleotide of the sgRNA, several studies have reported the potential off-target effects that gives rise to chromosomal rearrangements and can induce mutations. These off-target effects include incorporation of DNA mismatches in PAM-distal part of the sgRNA sequence [32, 58, 139–141]. Some strategies to avoid or at least minimize off-target effects of CRISPR/Cas9 editing include (i) choosing unique target sites that lack homology to any other region of the genome, (ii) modifying Cas9 by replacing wild type Cas9 with dCas9, (iii) fusing dCas9 with FokI nuclease (fCas9) which has higher sequence targeting specificity, (iv) modifying sgRNA by truncating the 3′end of sgRNA, and (v) reducing the concentration of Cas9-sgRNA delivered to cells [133].

## Future direction

CRISPR-based technologies have turned out be indispensable for scientific advancements. This is due to its wide applicability in both basic research in understanding fundamentals questions about how genes work as well as in the development of biotherapies for complex diseases like cancer. However, there are still several challenges associated with this technology that need to be addressed. For instance, the large size of Cas9 makes it difficult to package the protein in low immunogenic AAV vectors used in vivo and in vitro gene delivery. Also, Cas9 from *S. aureus* and *S. pyogenes* has been shown to cause infectious diseases in humans. One possible strategy to overcome this issue is to redesign Cas9 or use a different bacterial protein that is able to escape the host immune response. Intellia Therapeutics has developed a lipid-nanoparticle based CRISPR/Cas9 delivery system, which is enough to edit genes in rodents and non-human primates. There are also ethical concerns about this technology. With the reported birth of the “CRISPR babies” in November 2018, scientists have to collectively respond to the challenge of CRISPR potentially ushering in the era of genetic inequality and its long-term ramifications. Although these challenges persist, CRISPR-based technologies hold immense potential and are a great addition to the genome editing toolbox for the development of biotherapies that can improve patient outcomes in the future.

## Abbreviations

AML: acute myeloid leukemia
AAVs: adenoassociated viruses
fCas9: Cas9 with FokI nuclease domain
CAR-T: chimeric antigen receptor
CML: chronic myelogenous leukemia
CRISPR: clustered regularly interspaced short palindromic repeats
CRISPRa: CRISPR activator
Cas9: CRISPR-associated nuclease 9
CRISPRi: CRISPR interference
crRNA: CRISPR RNA
CDK6: cylin-dependent kinase 6
CTLA-4: cytotoxic T lymphocyte-associated protein 4
DSBs: DNA double strand breaks
DSBR: DNA double strand break repair
GOF: gain-of-function
HCC: hepatocellular carcinoma
HDR: homology directed repair
HR: homologous recombination
Indel: insertion/deletion
LBC: lobular breast carcinoma
LOF: loss-of-function
NHEJ: non-homologous end joining
dCas9: nuclease-dead protein
PTPN2: phosphatase non-receptor type 2
PML: promyelocytic leukemia
sgRNA: single-guide RNA
TRAC: T-cell receptor alpha constant
TOP2A: topoisomerase II
tracrRNA: trans-activating crRNA
TALENs: transcription activator-like effector nucleases
ZFNs: zinc finger nucleases
